# ICTV Virus Taxonomy Profile: *Metaviridae*

**DOI:** 10.1099/jgv.0.001509

**Published:** 2020-10-13

**Authors:** Carlos Llorens, Beatriz Soriano, Mart Krupovic, ICTV Report Consortium

**Affiliations:** ^1^​ Biotechvana, Scientific Park University of Valencia, 46980, Paterna, Valencia, Spain; ^2^​ Archaeal Virology Unit, Institut Pasteur, 75015 Paris, France

**Keywords:** Metaviridae, ICTV, taxononmy, retrotransposon

## Abstract

*Metaviridae* is a family of retrotransposons and reverse-transcribing viruses with long terminal repeats belonging to the order *Ortervirales*. Members of the genera *Errantivirus* and *Metavirus* include, respectively, Saccharomyces cerevisiae Ty3 virus and its Gypsy-like relatives in drosophilids. This is a summary of the International Committee on Taxonomy of Viruses (ICTV) Report on the family *Metaviridae,* which is available at ictv.global/report/metaviridae.

## Virion

**Fig. 1.**
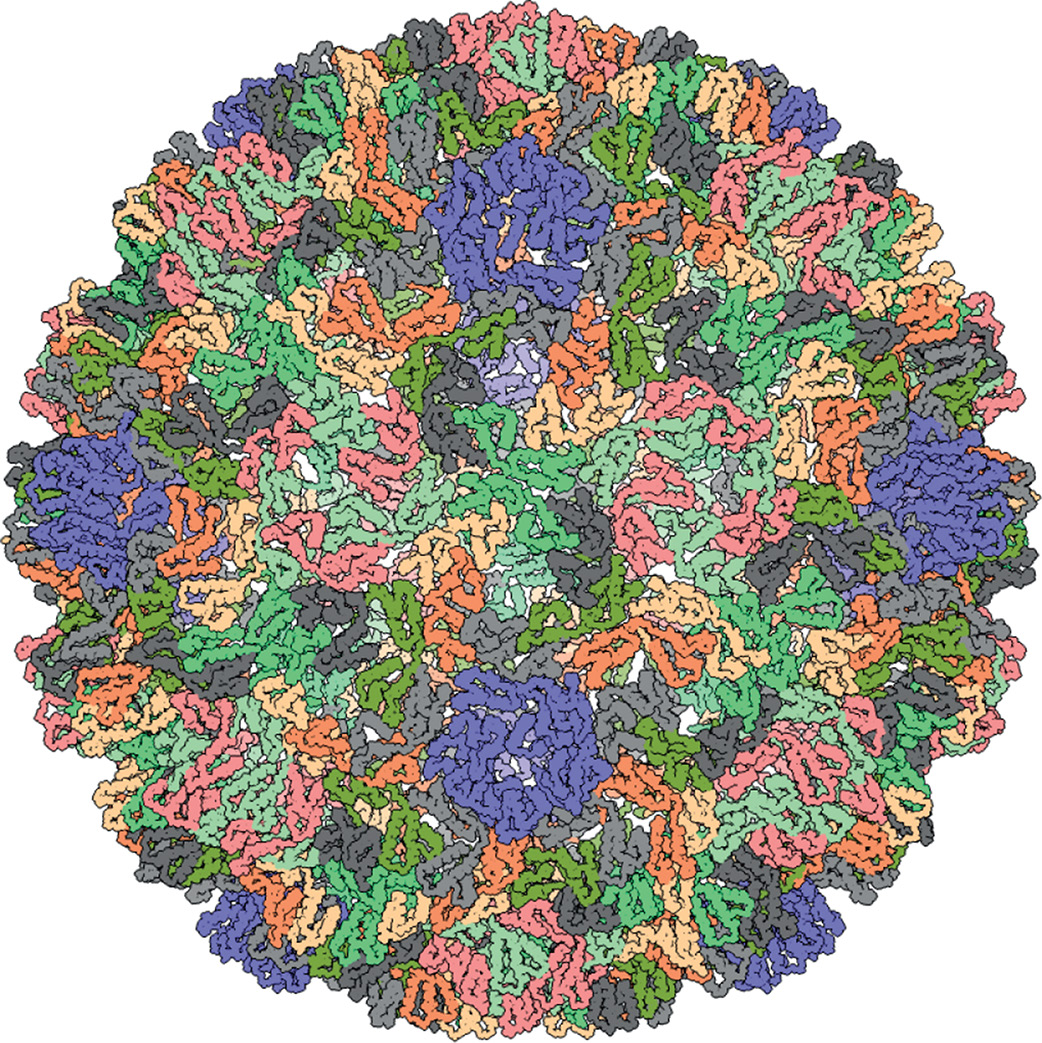
Three-dimensional reconstruction of a VLP of Saccharomyces cerevisiae Ty3. Diameter about 47 nm. PDB id: 6R24 [1].

The morphology of virus-like particles (VLPs) is poorly characterized; a high-resolution structure is only available for Saccharomyces cerevisiae Ty3 virus (Table 1, Fig. 1). Saccharomyces cerevisiae Ty3 VLPs are icosahedral (*T*=9) and built from a capsid protein, which is homologous to the corresponding protein of retroviruses and other members of the order *Ortervirales* [1–3]. Most VLPs do not appear to be infectious extracellularly, although Drosophila melanogaster Gypsy virus does generate infective VLPs [4]. By analogy with members of the family *Retroviridae*, VLPs are thought to contain two copies of the viral RNA genome, complexed with the nucleocapsid protein, cellular tRNAs involved in the priming of reverse transcription, reverse transcriptase and integrase. Virions of Drosophila melanogaster Gypsy virus also contain the envelope glycoprotein responsible for recognition and binding of VLPs to host cells and fusion of the viral and cellular membranes [5].

**Table 1.** Characteristics of members of the family *Metaviridae*

Typical member:	Saccharomyces cerevisiae Ty3 virus (M34549), species *Saccharomyces cerevisiae Ty3 virus*, genus *Metavirus*
Virion	Virions are icosahedral (*T*=9) and might be enveloped
Genome	Two identical copies of linear single-stranded, positive-sense RNA
Replication	Replication by reverse-transcription primed with a host-encoded tRNA
Translation	Genomic RNA is translated into one or more polyproteins
Host range	Fungi, plants and animals
Taxonomy	Realm *Riboviria*, kingdom *Pararnavirae*, phylum *Artverviricota*, class *Revtraviricetes*, order *Ortervirales*, family *Metaviridae*; the genera *Errantivirus* and *Metavirus* include >30 species

## Genome

**Fig. 2.**
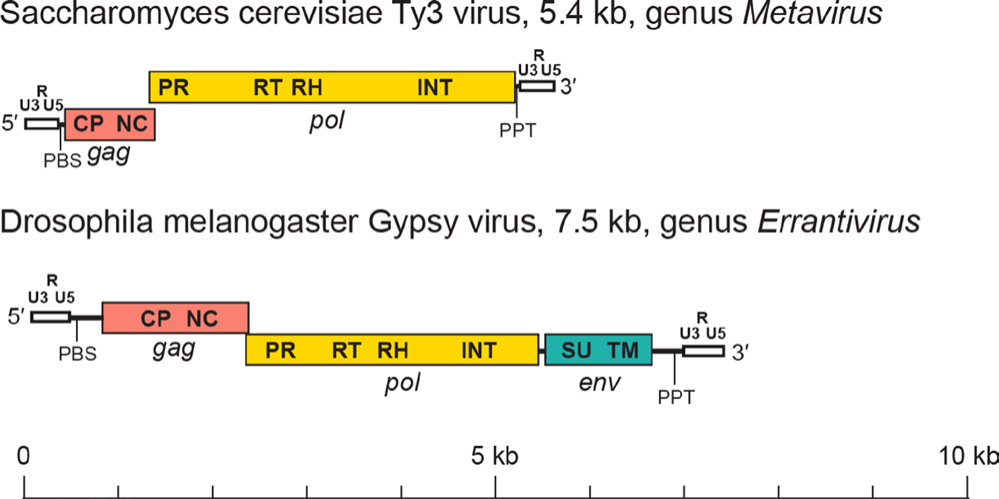
Metavirus genome structure. LTRs (white) are labelled with the U3, R and U5 regions. Other labels are PBS (primer binding site), PPT (polypurine tract), *gag* (pink) with its capsid (CP) and nucleocapsid (NC) domains, *pol* (yellow) with its protease (PR), reverse transcriptase (RT), ribonuclease H (RH) and integrase (INT) domains, and *env* (blue-green) with its surface (SU) and transmembrane (TM) domains.

The genome of a canonical member of the *Metaviridae* has an internal region that may range from 3 kb to > 15 kb flanked by two homologous non-coding sequences called long terminal repeats (LTRs) (Fig. 2). A canonical LTR has three subregions, U3-R-U5, which are analogous to those of retroviruses. ‘U3’ (200–1200 nt) contains the promoters; ‘R’ is repeated on each end of the transcript; and ‘U5’ (75–250 nt) constitutes the first portion of the reverse-transcribed genome. The internal region is delimited by two small motifs: an 18 nt sequence downstream of the 5′-LTR (the primer binding site, PBS) and about ten A/G residues located upstream of the 3′-LTR (the polypurine tract, PPT). The internal region may have one (*gag-pol*), two (*gag* and *pol*) or three (*gag*, *pol* and *env*) genes. The *gag* gene encodes domains for the capsid and the nucleocapsid proteins, whereas the *pol* gene includes domains for the protease, reverse transcriptase, ribonuclease H and integrase proteins. Where present, envelope (Env) polyproteins contain transmembrane and surface domains and are encoded downstream of the integrase domain [6, 7].

## Replication

Members of the family *Metaviridae* replicate via reverse transcription within intracellular VLPs. The cellular tRNA packaged in the VLP anneals to the RNA genome in the PBS region complementary to the 3′-end of that tRNA and is used by reverse transcriptase as an initiation primer. The proviral cDNA is imported into the nucleus, followed by integration into a chromosomal target site by the integrase.

## Taxonomy

Current taxonomy: ictv.global/taxonomy. The genera *Errantivirus* and *Metavirus* differ by the presence or absence of the *env* gene*,* respectively. However, this criterion is inconsistent with current knowledge of the evolutionary history of the family *Metaviridae*. While the genus *Errantivirus* constitutes a monophyletic clade, members of the genus *Metavirus*, form distinct clades showing polyphyletic relationships with each other. Furthermore, not all errantiviruses have *env* genes and, conversely, certain members of the genus *Metavirus* have *env* genes. A revision of the *Metaviridae* is required.

## Resources

Full ICTV Report on the family *Metaviridae*: ictv.global/report/metaviridae

Gypsy Database (GyDB) devoted to viruses and mobile genetic elements: http://gydb.org
